# The Potential Role of Nutrition in Overtraining Syndrome: A Narrative Review

**DOI:** 10.3390/nu15234916

**Published:** 2023-11-24

**Authors:** Maria Ester la Torre, Antonietta Monda, Antonietta Messina, Maria Ida de Stefano, Vincenzo Monda, Fiorenzo Moscatelli, Francesco Tafuri, Emma Saraiello, Francesca Latino, Marcellino Monda, Giovanni Messina, Rita Polito, Domenico Tafuri

**Affiliations:** 1 Department of Clinical and Experimental Medicine, University of Foggia, 71122 Foggia, Italy; ester.latorre@unifg.it (M.E.l.T.); maria.destefano@unifg.it (M.I.d.S.); giovanni.messina@unifg.it (G.M.); 2 Department of Experimental Medicine, Section of Human Physiology, Unit of Dietetics and Sports Medicine, University of Campania “Luigi Vanvitelli”, 80138 Naples, Italy; antoniettamonda99@gmail.com (A.M.); marcellino.monda@unicampania.it (M.M.); 3 Department of Precision Medicine, University of Campania “Luigi Vanvitelli”, 80138 Naples, Italy; antonietta.messina@unicampania.it; 4 Department of Economics, Law, Cybersecurity, and Sports Sciences, University of Naples “Parthenope”, 80131 Naples, Italy; vincenzo.monda@uniparthenope.it (V.M.); emma.saraiello@uniparthenope.it (E.S.); domenico.tafuri@uniparthenope.it (D.T.); 5 Department of Human Sciences, Telematic University Pegaso, 80100 Naples, Italy; fiorenzo400@gmail.com (F.M.); francesca.latino@unipegaso.it (F.L.); 6 Heracle Lab Research in Educational Neuroscience, Niccolò Cusano University, 00166 Roma, Italy; francesco.tafuri@unicusano.it

**Keywords:** overtraining syndrome (OTS), overreaching, physical activity, nutrition, dietary intake

## Abstract

Competition between athletes and an increase in sporting knowledge have greatly influenced training methods while increasing the number of them more and more. As a result, the number of athletes who have increased the number and intensity of their workouts while decreasing recovery times is rising. Positive overtraining could be considered a natural and fundamental process when the result is adaptation and improved performance; however, in the absence of adequate recovery, negative overtraining could occur, causing fatigue, maladaptation, and inertia. One of the earliest forms of fatigue is overreaching. It is considered to be an accumulation of training that leads to reduced sports performance, requiring days or weeks to recover. Overreaching, if followed by adequate recovery, can lead to an increase in athletic performance. Nonetheless, if overreaching becomes extreme, combined with additional stressors, it could lead to overtraining syndrome (OTS). OTS, caused by systemic inflammation, leads to central nervous system (CNS) effects, including depressed mood, further inflammation, central fatigue, and ultimately neurohormonal changes. There are therefore not only physiological, biochemical, and immunological but also psychological symptoms or markers that must be considered, independently or together, being intrinsically linked with overtraining, to fully understand OTS. However, to date, there are very few published studies that have analyzed how nutrition in its specific food aspects, if compromised during OTS, can be both etiology and consequence of the syndrome. To date, OTS has not yet been fully studied, and the topic needs further research. The purpose of this narrative review is therefore to study how a correct diet and nutrition can influence OTS in all its aspects, from prevention to treatment.

## 1. Introduction

In the competitive sport sector, it is natural for athletes to increase their training loads to improve performance [1]. This increase in muscular work is tolerated only through time intervals consisting of muscle recovery and rest and periodization of training [2]. Overtraining syndrome (OTS) occurs following a prolonged period of overreaching (OR) combined with additional stressors [3], where OR is defined as an accumulation of training load leading to decreased performance and with days to weeks for muscle recovery [3,4]. OR can be subdivided into functional OR (FOR) and non-functional OR (NFOR), where FOR refers to the positive improvement in fitness and performance since recovery has occurred, while NFOR is associated with the impairment of performance and fitness due to a lack of muscle recovery [4,5,6]. The difficulty, therefore, is in understanding whether an athlete has achieved OTS or NFOR status. The difference between FOR, NFOR, and OTS depends on the time necessary for the individual and the body to recover; in fact, OR generally requires days or weeks, NFOR requires weeks or months, and OTS can require months or even years of recovery [3,4,5,6,7]. Therefore, OTS can be defined, as reported by Sims et al. an accumulation of stress whether or not due to training, which results in a long-term decrease in performance capacity with or without associated physiological or psychological signs and symptoms of overtraining, in which recovery of performance capacity may take several weeks or months [8]. 

**Figure 1:**
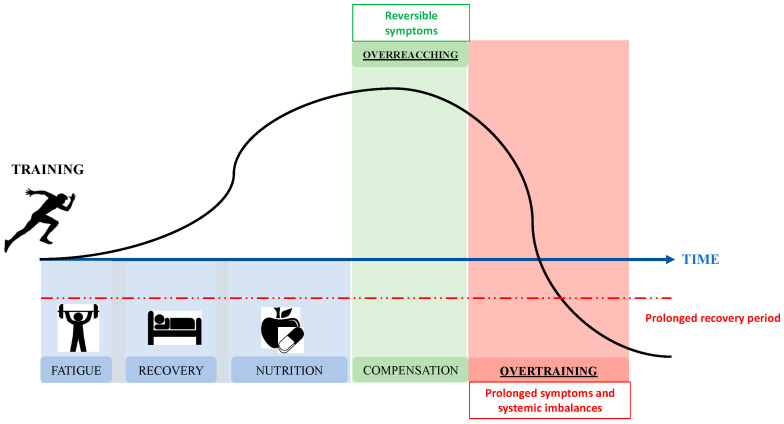
Process of development of overtraining syndrome in athletes.

The causes of OTS can be varied, such as systemic inflammation and consequent effects on the central nervous system including neurohormonal changes, depressed mood, and a sense of fatigue [9] (Figure 1). Furthermore, other causes of the onset of OTS could be depletion of glycogen and tissue trauma, and increased levels of cytokines but also depletion of glutamine in the muscles with consequent muscle pain, weight loss, and mood changes or frequent illnesses which can accompany the decrease in performance [10,11,12,13,14].

Epidemiologically, OTS is extremely rare, but given the difficulty in diagnosis, exact data on prevalence and incidence are currently lacking [15,16]. Epidemiological analyses have found that episodes of NFO and OTS can occur during the athlete’s career; for example, according to the literature, about 35% of adolescent swimmers had been overtrained at least once in their career [17], 5–30% had experienced staleness [16], and approximately 60% of elite male and female runners experienced NFOR symptoms compared to 33% in non-elite runners [18]. Furthermore, the risk of OTS appears to be significantly increased in individual sports and low-intensity sports [19]. Therefore, overtraining is still an underestimated phenomenon, with consequent lower interest in the scientific literature in this regard; indeed, to date, OTS has not been fully studied and the topic currently requires further scientific research. The purpose of this narrative review, therefore, is to study and explore how correct nutrition and integration can influence and modulate OTS in all its aspects, from prevention to treatment, to reduce the onset of the phenomenon, contributing to a primary prevention perspective, in line with global standards.

## 2. Pathophysiology of OTS

In general, regular exercise of mild to moderate intensity is used as a non-pharmacological intervention strategy to prevent various pathological medical conditions such as type 2 diabetes, insulin resistance, pulmonary and cardiovascular diseases, colon cancer and breast cancer, dementia, and depression, and to improve the symptoms [20,21,22,23,24,25,26]. Despite this, it is necessary to highlight the paradox of the overtrained athlete; as already stated, excessive training can aggravate the decline in sports performance [20,27]. Numerous theories on the causes of this phenomenon have been proposed and studied.

### 2.1. Cytokine Hypothesis

In addition to the association between overtraining and skeletal muscle injury that was established about two decades ago, the cytokine hypothesis was introduced, which integrates and explains most of the signs and symptoms that appear to be linked to the decline in sports performance induced by excessive training [11,21,28]. Therefore, the hypothesis states that high-load training associated with insufficient recovery could induce musculoskeletal trauma and subsequently determine the production and release of cytokines and interleukins such as IL-1 beta, IL-6, and TNF-alpha with subsequent multi-organ interaction [11,12]. This theory has been extensively studied through various works in multiple fields. For example, elevated levels of IL-6 and TNF-alpha in skeletal muscle have been shown in mouse studies to be related to impairment of the insulin signaling pathway, resulting in insulin resistance, muscle atrophy, and activation of endoplasmic reticulum (ER) stress [20,29]. Furthermore, high levels of cytokines such as IL-1 beta, IL-6, and TNF-alpha, derived from peripheral inflammatory stimuli, at the hypothalamic level are linked to an increased restriction of food intake among the cytokines. TNF-alpha plays a fundamental role in reduced food intake and increased cellular catabolism, causing difficulty in increasing body weight [30,31,32,33,34]. Furthermore, based on the theoretical correlation between inflammation and ER stress [29], a study by Pinto et al. states that the activation of hypothalamic ER stress is present above all following excessive training [35]. These data strengthen the theory of proinflammatory cytokines, as they could act as an initial stimulus in the central nervous system, inducing some maladaptation soon after a period of excessive training, affecting physical performance, food restriction, and cellular catabolism [36]. Finally, the inflammatory role of cytokines may also be linked to the development of pathological cardiac hypertrophy, due to the increase in IL-6 [31]. Indeed, recently, there has been an increase in IL-6 protein levels, a reduction in the activation of AMPK-activated protein kinase, mTOR, and ribosomal protein rpS6, and signs of left ventricular fibrosis in mice [37]. The results suggest that excessive training can induce signs of pathological cardiac hypertrophy, thus contributing to impairment and reduction in physical performance [37]. Thus, it is difficult for athletes to manage and recover by modulating overtraining at a systemic level due to the proinflammatory action of cytokines. 

### 2.2. Glutamine Hypothesis

The production of cytokines is not the only reason for the onset of overtraining syndrome; there is also a relationship between hormones and overtraining syndrome, the latter of which can lead to complex hormonal dysfunctions [3]. Nonetheless, the diagnosis of OTS is complicated and can only be made after the exclusion of other more common medical conditions. Physiological, biochemical, and immunological markers for overtraining have received much attention in recent years and could be of potential use if routinely measured in the laboratory as a medical aid in preventing overtraining [38]. Glutamine is among the various molecules that could be used as biomarkers of overtraining. Glutamine is an essential amino acid for the function of immune cells, in particular [4,12,39]. It also plays a key role in DNA and RNA synthesis, nitrogen transport, and acid–base balance and gluconeogenesis [4,13,39]. The decrease in glutamine after physical exercise could be responsible for an increase in the incidence of infections of the respiratory system and of the upper respiratory tract (URTI) in overtrained athletes [4,11,40]. Prolonged training or repeated periods of high-intensity training could transiently reduce plasma glutamine concentrations, resulting in greater sensitivity to URTIs [11,13,39]. As described in the cytokine theory, plasma glutamine also plays a key role in various processes controlled by cytokines [41]. Glutamine is a molecular precursor of the synthesis of inflammatory proteins which are upregulated with overtraining [12]. Furthermore, systemic inflammation, given by the increase in proinflammatory cytokines, induces a catabolic state with a consequent increase in both glucose and protein metabolism. Therefore, glutamine is a crucial element in gluconeogenesis. Furthermore, alterations in the hypothalamic–pituitary–adrenal (HPA) and gonadal (HPG) axis, following some studies in the literature, appear to be responsible for OTS. In particular, endurance athletes might show subtle changes in HPA axis function and thus show alterations in the levels of cortisol, ACTH, testosterone, and other hormones [42,43,44,45]. In a study by Barron et al., an adrenocortical deficit was also highlighted in athletes with overtraining syndrome [44]. That is, the data show that growth hormone, prolactin, and ACTH responses to insulin-induced hypoglycemia were lower in overtrained athletes than in healthy, well-trained controls. Unfortunately, however, current data and research are in some cases inconsistent regarding the patterns of these hormonal changes, as they depend on various factors including individual training capacity, intrinsic vulnerability to stressful factors, and other hormonal levels [45]. 

### 2.3. Central Fatigue Theory

OTS is often characterized by mood, sleep–wake cycle, and behavioral disturbances [15,43,46,47]. The neurotransmitter involved in the regulation of these mechanisms is serotonin (5-HT); 5-HT alterations could trigger OTS [3,12,46,47,48]. Central fatigue theory hypothesizes that OTS is caused by an increase in the synthesis of 5-hydroxytryptomine (5-HT) in the central nervous system (CNS). Physical exercise decreases the levels of branched-chain amino acids (BCAA) due to an increase in their oxidation into glucose, favoring the entry of tryptophan into the brain and the subsequent conversion into 5-HT as both BCAAs and tryptophan use the same transporter to cross the blood–brain barrier [48]. Therefore, a decrease in plasma BCAAs with a consequent increase in plasma tryptophan lead to an increase in CNS tryptophan levels [43,48]. 5-HT derives from tryptophan. Budgett et al. studied how the administration of serotonin reuptake inhibitors to athletes increases the levels of 5-HT with consequent reduction in the athletes’ performance [48], while the integration of branched-chain amino acids therefore reduced 5-HT production and resulted in increased physical and mental energy [49]. 

### 2.4. Glycogen Hypothesis

Another phenomenon that could interfere with an alteration of the central neurotransmitters involved in fatigue during OTS could be a reduction in muscle glycogen due to overtraining [50]. Glycogen is the key energy source during moderate to vigorous exercise [50]. Low muscle glycogen also results in an increase in oxidative processes and a decrease in total concentrations of BCAA, as reported, leading to central fatigue [50]. Some studies report that, during OTS, there are lower amounts of lactate in the blood in overtrained athletes compared to well-trained athletes [51,52]. Therefore, post-exercise blood lactate measurement could be used as a tool to identify athletes at risk of overtraining, especially if such testing were performed on a regular basis [51,52]. 

### 2.5. Autonomic Nervous System Hypothesis

Certainly, an imbalance in the autonomic nervous system, reducing sympathetic activation and parasympathetic dominance, could lead to some symptoms of OTS such as decreased performance, fatigue, depression, and bradycardia, but also to a reduced nocturnal urinary excretion of catecholamines [4,12,43]. Generally, catecholamine excretion decreases with increasing training and fatigue, returning to baseline levels during the recovery period [4,53]. A reduced organ sensitivity to catecholamines could therefore be an indicator of symptoms of reduced sympathetic activation [43]. Furthermore, studies have stated that the effects of intense training on autonomic nervous system control may be reversible, even after a week of rest [54]. Heart rate variability (HRV) monitoring could therefore be an indicator of autonomic function and a predictor of overtraining [45,55]. 

### 2.6. Oxidative Stress Hypothesis

Intense physical exercise could generate reactive oxygen species (ROS) and consequently increase oxidative stress [56]. ROS are related to post-exercise inflammatory response mechanisms and to the propagation of muscle damage with promotion of ROS-induced infiltration of neutrophils and macrophages into muscle [57,58]. Neutrophils and macrophages generate a free radical such as superoxide, which in turn can be converted into hydrogen peroxide, which is then able to react with the superoxide in the presence of a transition metal to form hydroxyl radicals [58]. Furthermore, the typical markers of protein oxidation and the total oxidizing capacity were elevated, while the levels of antioxidants such as glutathione and coenzyme Q10, and γ-tocopherol and carotenoids were decreased [59,60,61]. Alterations in redox homeostasis have also been reported in individuals with OTS; however, information on ROS generation in OTS needs further research. The identification of common biomarkers for overtrained athletes could allow an adequate intervention to prevent the progression of fatigue towards a more severe stage of overtraining syndrome [59,60,61].

## 3. Dietary Intake in OTS

Overtraining syndrome, and therefore an imbalance between training and recovery, can be aggravated or implied by improper nutritional intake [62,63,64]. Nutrition therefore plays a fundamental role in maintaining correct body homeostasis, as an imbalance in the quantities and proportions of macro- and micronutrients, but also an incorrect state of hydration, could affect correct energy intake, the maintenance of normal physiological functions, and good body composition, necessary for recovery [5]. However, there are few studies in the literature on the relationship between nutrition and OTS. Some of the published studies state that inadequate nutrition, pathologies, and stress factors and sleep disorders [65] can lead to dysfunctions of the normal metabolic processes involved in immune, inflammatory, neurological, hormonal, and metabolic responses [65]. For example, it has already been demonstrated that overtraining syndrome can reduce an athlete’s appetite, producing a counterproductive effect, since if the athlete presents continuous caloric deficits during training, these could elevate both the stress hormone and the response and production of cytokines during physical exercise, limiting the athlete [11,12,66,67,68,69,70]. Furthermore, fatigue, injury, immunosuppression, and decreased performance can occur not only when energy expenditure regularly exceeds daily caloric intake but also when hydration is insufficient [70,71,72]. In fact, incorrect hydration could have a negative effect not only on performance but also on the metabolism of some macronutrients, such as glycogen, which can only be stored in the muscle when bound to water in a ratio of 1:3 g [72]. It has been found that even low levels of dehydration can compromise both the athlete’s exercise capacity and the cardiovascular and thermoregulatory responses [73]. Optimal performance, therefore, is possible only when dehydration as well as hyperthermia is minimized by ingesting large volumes of fluids during exercise. Therefore, the consumption of liquids in volumes similar to the amount of sweat lost results in the maintenance of important physiological functions and significantly improves exercise performance, even during exercise lasting only 1 h. Carbohydrate ingestion also improves exercise performance, an effect that is independent of and additional to the prevention of dehydration [73]. A study conducted by Candegiani et al. demonstrated how an adequate daily caloric intake, regardless of the composition of macronutrients, can have positive bodily influences, for example, raising salivary cortisol 30 min after waking up, improving the speed and quality of muscle recovery, and increasing the elimination rate of muscle recovery markers, mainly creatine kinase (CK) and lactate [74]. Therefore, an increase in caloric intake could lead to a higher quality of sports performance by positively influencing both muscle recovery and the hormonal environment.

### 3.1. Dietary Fat

High levels of physical activity and an inadequate or unbalanced nutritional intake are implicated in imbalances, above all hormonal [63,75,76,77]. Therefore, in addition to a correct daily caloric intake, it is also important to maintain an adequate number of macronutrients. Each macronutrient has its own precise role, for example, the daily lipid component plays important physiological roles, especially in the construction of cell membranes, as fuel for exercise, as antioxidants, and as precursors of some hormones [78]. Recent studies have shown that low-fat diets impair sports performance [79,80,81]. For example, a high-fat diet has been shown in some studies to potentially reduce plasma levels of pro-inflammatory cytokines such as IL-6, IL-1 and TNF-α, due to the anti-inflammatory action of omega-3 fatty acids [82,83,84,85,86]. Furthermore, increasing the amount of lipids in the diet can increase muscular endurance without the appearance of adverse effects, especially on plasma levels of cortisol, interferon-γ, and PGE2 [78]. Some studies state that reducing the fat content in the diet could reduce circulating testosterone levels, implicated in dysregulation of the hypothalamic–pituitary–thyroid (HPTA) axis negatively affecting health and physical performance [84]. A study by Hamalainen et al. reports that reducing dietary fat from a daily intake of approximately 40% of energy to a daily intake of less than 25% of energy resulted in an approximately 15% decrease in total testosterone levels compared to a diet with a high percentage of fat, about 41% of energy intake, and a reduction in polyunsaturated fatty acids [83,85,86]. Despite this, it should be emphasized that a diet rich in fats is highly discouraged due to negative effects on the cardiovascular system, which is why the total quantity of fats in the daily diet must comply with the guidelines provided by the World Health Organization (WHO), in which the percentage of total lipids must not exceed 30% of total calories, combined with an intake of saturated fatty acids of less than about 10%, and an intake of lower trans fatty acids at 1%, favoring the consumption of unsaturated fatty acids [87,88,89,90]. Precisely for this reason, new research has focused on how to improve sports performance without increasing the fat component beyond the recommended percentages, and in this case, ketone ester supplementations (KEs) seem to be a valid alternative. Interestingly, in some studies, KEs significantly inhibited the onset of fatigue symptoms while improving the training load tolerated by the athlete, especially resistance exercise performance, and increasing total energy intake. Furthermore, KEs counteracted training-induced sympathetic overdrive, as also evidenced by stable nocturnal catecholamine production. KEs also presented a marked and positive impact on tachycardia following exercise, thus demonstrating that KEs are a powerful nutritional strategy that prevents the development of physiological symptoms of overtraining and determines their overcoming [91]. Currently, medium-chain triglyceride (MCT) supplements as a source of fat and as an energy source have also gained greater attention in the sports field. MCTs could directly enter the mitochondrial level and be used for energy production through the beta-oxidation process [92]. This process would provide the athlete with an immediate source of fat that can be used for energy purposes, thus saving glycogen consumption [36]. Although there are studies that state that MCTs show improved athletic performance, other studies show side effects such as gastrointestinal disorders when taking MCTs compared to carbohydrates [92]. Further research is therefore necessary in the field of sports nutrition supplementation in this area.

### 3.2. Dietary Protein

The protein component, on the other hand, is essential for the maintenance and growth of muscle tissue [78]. Following high-intensity workouts, the ratio between protein degradation and synthesis increases, resulting in a greater consumption of dietary protein [78,93]. Therefore, there is a linear relationship between protein intake and loss of lean mass during periods of negative energy balance, so a good protein supplementation proves to be an effective strategy to mitigate the loss of lean body mass [94,95]. An imbalance in the mechanisms involved in the defense of the individual has been observed due to moderate and severe protein imbalance [82,96]. It has been reported that diets low in protein (<20%) or, on the contrary, abundant in protein (>60%) have been shown to impair phagocytosis activity and the production of interleukins such as IL-2, resulting in potential damage to the T cell system [82]. Glutamine plays a fundamental role in protein composition, as previously mentioned. Glutamine is mainly used as fuel for lymphocytes and macrophages for the synthesis of DNA and RNA [13,97,98,99,100]. Intense exercise can lead to depletion of skeletal muscle stores; therefore, it has been hypothesized that glutamine depletion may be linked to impaired immunity [99,100]. As stated in one study, a low protein intake can be responsible for protein loss, but in reality, even a high protein intake can be harmful and cause an approximately 25% decrease in the amount of plasma glutamine due to an increase in renal absorption [78]. Furthermore, the dietary protein component is an important determinant of body characteristics; in fact, it is positively correlated with parameters such as metabolism and body composition, and with an increase in the basal metabolic rate (BMR), fat oxidation, muscle mass, and consequent hydration. Protein intake is significantly but inversely related to extracellular water, i.e., it protects against water loss, thus preventing edema [74,101,102,103,104]. Finally, growth hormone (GH) and insulin-like growth factor-1 (IGF-I) appear to be related to healthy diet and lifestyle, while they appear to be impaired during OTS [105,106]. IGF-1 mediates many of the actions of GHs, which regulate growth processes, maintenance above all of lean mass but also bone mass, cellular differentiation, and mitochondrial homeostasis, a process that is, in turn, key to the metabolic processing of carbohydrates, fats, and amino acids (AAs) [107,108,109]. During a fasted condition, GH primarily inhibits and reduces protein degradation by stimulating its synthesis in muscle and other tissues [110]. This defense mechanism, to all intents and purposes, could also be mediated by IGF-I, considering that chronic GH exposure increases hepatic IGF-I production [111]. In order, therefore, to maintain protein homeostasis and to support and/or prevent OTS, protein requirements for healthy endurance athletes have been estimated at approximately 1.2-1.4 g/kg and slightly higher for endurance training athletes up to about 1.7 g/kg [112,113]. The acceptable macronutrient distribution range (AMDR) states that proteins can vary from 0.8 g/kg to 2.5 g/kg based on individual needs, converted in percentage from 10% to 35% of energy, but the increase in protein requirement and intake to date remains controversial [114,115]. As regards the integration of BCAAs, whose imbalance appears to be a cause of the emergence of overtraining, currently, in this case the results are not yet convincing. As stated by Gastmann et al., BCAAs, if taken with an adequate dose of carbohydrates during prolonged physical exercise, would not seem to determine improvements in OTS. Furthermore, high doses of ingested BCAAs could increase the secondary production of ammonia, leading to further premature fatigue, as BCAAs are considered the main donors of amino groups at a muscular level. Overall results in this field are currently inconclusive and require more controlled experimental research [116]. Finally, regarding the use of creatine as a sports protein supplement, currently, despite its widespread use in this field, studies and research are still needed to explore the topic in more depth. From what has emerged in the literature, creatine could have a positive effect on muscle recovery, muscle hypertrophy, and strength. In a study by Wang et al. and Ribeiro et al., the use of a higher dose of approximately 20 g per day (period of approximately 5 days), and a subsequent maintenance dose of approximately 3 g per day (51 days), combined with resistance training, led to hypertrophy of the skeletal muscle mass, less fatigue, and reduction in creatine kinase in the creatine-treated group compared to the control group [117,118].

### 3.3. Dietary Carbohydrate

In addition to lipids and proteins, carbohydrates, according to numerous studies conducted in recent years, play a role as the main macronutrient to support and improve physical performance [119]. Carbohydrates, therefore, play the role of primary fuel, especially for the first 60–90 min of sports activity in the form of muscle and liver glycogen [120]. Similarly, after physical activity, the intake of carbohydrates supplies the muscle cells with the fuel necessary to begin the muscle reconstruction processes. Using the energy gained from burning carbohydrates, the muscles can absorb amino acids from the bloodstream, initiating protein synthesis [119]. Furthermore, carbohydrates stimulate the production and release of insulin, an anabolic hormone that plays a key role both in protein synthesis and in the reduction of protein breakdown [120]. During exercise, muscle cell injury often occurs with a characteristic increase in plasma creatine kinase activity and a consequent increase in post-exercise cytokine release [121]. Furthermore, a reduction in blood glucose levels may be linked to activation of the hypothalamic–pituitary–adrenal (HPA) axis, increased release of adrenocorticotropic hormone (ACTH) and consequently cortisol, increased GH levels, a decrease in insulin levels, and highly variable levels of adrenaline [122,123]. Several studies in the literature suggest that carbohydrates have effects on the production of IL-6 and the IL-1 receptor antagonist (IL-1ra) involved in the inflammatory cascade response to heavy exertion, reducing their plasma levels and altering the release [98,124,125]. In addition, the consumption of carbohydrates during physical activity appears to attenuate the increase in hormones such as cortisol and GH, limiting the degree of immunosuppression following physical exercise [123]. While increasing the amount of carbohydrates has been shown to have predominantly positive effects on the hormonal profile, however, excessive intake could have a pro-inflammatory role [126,127]. In fact, it could potentially induce a pathological increase in the action of aromatase, i.e., the enzymatic system responsible for the conversion of the androgen hormone, testosterone, into estrogen, estradiol, causing an imbalance in the testosterone–estradiol (T/E) ratio [101,102,104,126]. Furthermore, an excessive intake of carbohydrates could lead to an increase in lactate levels and a slight, but not significant, increase in neutrophils [126,127,128,129]. In conclusion, the deprivation of carbohydrates can determine reduced and/or delayed hormonal responses, which indirectly compromise the performance of athletes; therefore, to build excellent glycogen stores and help rebuild muscle tissue after physical activity, a rapid and adequate carbohydrate intake is required. If an intake of carbohydrates of less than about 5.0 g/kg/day has harmful effects on the hormonal level and on physical performance, in order to ensure that good muscle reserves and correct physiological functions are associated with it, carbohydrate intake should equal approximately 8 to 10 g/kg of body mass per day, which could represent 60 to 70% of total calories [74,106,127,130]. Furthermore, it is essential to evaluate the use of carbohydrate-based supplements during training and sports. Among the most used supplements are maltodextrins, which have been used in recent years. The effect of maltodextrins (polymers deriving from the hydrolysis process of starches [131]) during sports practice has been investigated in several studies, which however have presented conflicting results and opinions. From the results, it was noted that the use of the carbohydrate solution containing maltodextrins during training did not alter the blood concentrations of circulating leukocytes at an immune level [132], not resulting in an improvement in the immune system, while integration with a mixture of glutamine in combination with maltodextrins 2 h before training could be more effective in preventing a decrease in sports practice compared to the consumption of maltodextrin-based supplements or glutamine-based supplements alone [133]. Therefore, it is concluded that only carbohydrate supplementation in combination with glutamine peptide could improve the physical performance of athletes during competitions.

## 4. Micronutrients and OTS

Strenuous and intense physical activity can induce pathologies, injuries, and chronic fatigue, symptoms underlying overtraining syndrome, partly due to the toxicity of free radicals (FR) and to the altered response of the immune system [61,134]. In this context, in addition to macronutrients, micronutrients play a key role. A dietary imbalance may not provide an athlete with essential micronutrients, vitamins, and mineral salts for his or her health and physical performance [134].

**Figure 2:**
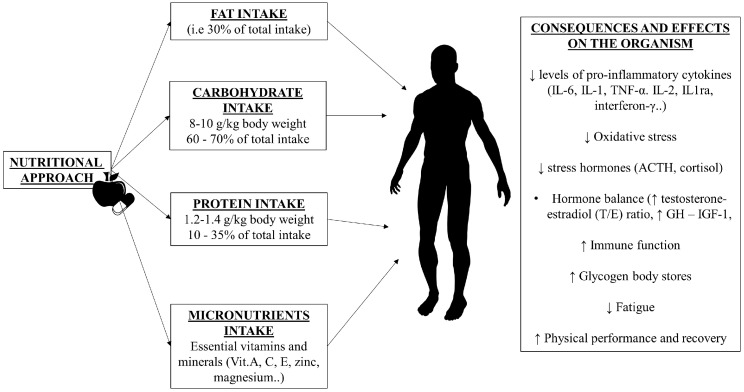
Basic notions and applications of the nutritional approach to preventing overtraining.

For example, vitamins C, E, and A play important antioxidant roles in the neutralization of oxygen free radicals (ROS) and in supporting the immune system [126]. Vitamin C, in addition to having an antioxidant effect, thus reducing the risk of colds and other viruses, improves the differentiation but also the proliferation of B and T lymphocytes by increasing the levels of circulating antibodies [135,136,137,138]. Furthermore, it also modulates post-exercise cytokine production [138]. Vitamin E is used against chronic stress associated with exercise, as it possesses antioxidant properties against ROS, protecting cells and tissues; it reduces PGE2 production and inhibits COX2 activity by reducing nitric oxide [138]. It improves the T immune system by modulating the Th1/Th2 balance [139]. Vitamin A, on the other hand, has a supportive action for various body functions, including reaction time, muscle recovery, and post-exercise protein synthesis, essential for muscle growth and recovery [140,141,142]. Studies state that the supplementation of vitamin C (500 mg), vitamin E (270 mg), and β-carotene (vitamin A—18 mg) significantly reduces the probability of infection after prolonged exercise from 40 to 15%, and that tocopherol, when taken with ascorbic acid, has optimal effects. Furthermore, ascorbic acid supplementation (600 to 1000 mg/day for 3 weeks) reduced symptoms of URTI in marathon runners [98,143]. Although the integration with different sources of antioxidants therefore provides greater protection of the tissues against the oxidative stress induced by physical activity, the data present in the literature are contradictory. Indeed, over-supplementation of some vitamins, when in intolerable doses, especially with a single antioxidant nutrient, is not strongly recommended [144,145,146]. For example, excessive doses of vitamin A could have toxic effects on the liver, vitamin E in excessive doses can cause increases in mortality risk factors, and high levels of vitamin C can act as a pro-oxidant and non-oxidant [145,146]. More evidence is needed to support high dosages of antioxidant vitamins. Vitamin D, on the other hand, predominantly plays a cooperative role in the synthesis of various hormones; moreover, there appears to be a direct relationship between vitamin D concentrations and the performance of athletes, such as speed, muscle tone, and grip strength, but it also improves the use of carbohydrates during exercise, providing athletes with more energy, which can help increase performance [82,147,148]. The addition of calcium together with vitamin D intake also shows a reduction in the rate of stress fractures [149,150]. However, calcium intake is usually lower than recommended doses in athletes’ diets, especially low-fat diets [151,152]. Precisely for this reason, in rare circumstances, especially if the diet is low in nutrients, physical activity could endanger bones [153]. Therefore, an adequate intake of vitamin D and calcium improves the athlete’s performance and physical form [153,154]. Vitamins of the B complex have a fundamental importance, as they contribute to maintaining both health and optimal performance in athletes, improving brain functioning, concentration, and quality of sleep, by regulating melatonin, and energy levels [154]. Among these, vitamin B12 and folic acid, in particular, have a key role and are essential above all for the normal production of red and white blood cells [134]. A possible lack of vitamin B12 or folic acid could determine a reduced proliferative response of lymphocytes, a reduction in the phagocytic and bacterial capacity of neutrophils, and therefore an altered immune response, as well as being, especially folic acid, crucial in lowering homocysteine levels, a risk factor for heart disease [155,156]. Finally, in addition to vitamins, minerals also play a fundamental role in sports [139]. For example, a low zinc intake could compromise the immune system, as it plays an important role in the development of T lymphocytes and resistance to infections. A good supply of zinc is supported by diets high in fat [78]. Furthermore, the immune function is sensitive to the availability of iron; its deficiency can reduce lymphocyte responses, resulting in IL-1 production by macrophages, reduced interferon production, and natural killer cell activity (NKCA) [82,157]. Iron is essential for producing red blood cells, which are necessary to transport oxygen to the muscles, and is involved in hormonal and physiological functions, particularly in women [158]. Restrictive or unbalanced diets, coupled with inadequate levels of exercise, could lead to anemia and other problems associated with low iron levels, such as fatigue, poor performance, and a reduced ability to perform physical activities [159,160]. Therefore, it is necessary to consume a high-quality and varied diet and foods that include sources rich in iron [161]. Selenium, on the other hand, plays a key role as an antioxidant and cofactor of glutathione peroxidase/reductase, affecting multiple aspects of the immune system, and may help improve an athlete’s performance and reduce inflammation [162]. Selenium deficiency is generally associated with elevated serum levels of C-reactive protein (CRP), a biomarker of inflammation [163]. Finally, potassium and magnesium are long-recognized key minerals in athletic performance [164]. Magnesium helps improve energy levels, increasing muscle performance and reducing exercise-induced fatigue [165]. Potassium, on the other hand, helps reduce the amount of lactic acid that is stored in the muscles, reducing the occurrence of cramps in athletes, and is also involved in the breakdown of carbohydrates, keeping energy levels high during physical activity, especially if intense [166,167]. In order not to provide too high a dose of the amounts of these two minerals, according to WHO guidelines, adults should not exceed 2000 mg sodium/day (Na) and have a minimum dose of 3510 mg potassium/day, as they are directly related [167,168,169]. The recommended dietary amount of magnesium is between 400 and 420 mg/day for males and between 310 and 320 mg/day for females [164]. Therefore, in summary, a multitude of physiological systems depend on micronutrients, which have a fundamental impact on the general health and performance of an athlete, without the need to exceed the values recommended by the guidelines, especially if the athlete consumes an adequate amount of nutrient-rich foods through a good dietary intake [170,171] (Figure 2). Unfortunately, many athletes do not meet the recommended requirements for most micronutrients, and therefore, more attention and correct supplementation are needed in this regard [171].

## 5. Discussion

Following a workout, inevitably, the body is urged to interrupt body homeostasis. Through correct training planning, therefore, an individually programmed workload progression associated with sufficient rest, and physical and functional adaptation for the athlete will be achieved, improving physical capacity and consequently performance [172]. However, excessive training and poor rest can undermine this process of physical adaptation and lead to overtraining syndrome, i.e., a condition with a marked decrease in performance despite rigorous training, systemic fatigue, a reduction in the defensive capabilities of the immune system, mood disorders, sleep disorders, biochemical and metabolic alterations, and finally, changes in physiological parameters such as heart rate at rest [6,11,12,41,46,51,88]. The cause of this syndrome is not only overtraining and poor rest but also the sum of additional stressors that could lead to its development. Poor or partially unbalanced nutritional intake could also be a contributing factor to OTS [126]. In general, adequate nutritional intake is involved in the various adaptive processes and above all for the recovery of the organism. Thus, an unbalanced diet could slow down the recovery process and the return to homeostasis [173]. Furthermore, to limit the risk of developing OTS, or to reduce the symptoms related to it, it is important to ensure that from a nutritional point of view there is the right amount of both macro- and micronutrients [173]. For example, an insufficient intake of carbohydrates and a decrease in muscle glycogen stores not compensated by diet could be a determinant of increased fatigue and poor performance with changes in the body’s hormonal response (increased release of catecholamines, changes in circulating levels of cortisol and testosterone–estradiol (T/E) ratio etc.) [173,174]. Furthermore, it is now well known that sustaining a workout with a low amount and availability of carbohydrates could accelerate the onset of fatigue [174]. In recent years, proteins have received greater attention, especially in the fields of sports nutrition and nutrition. Proteins and amino acids, especially those such as glutamine, have multiple and fundamental roles [175]. In fact, protein may be especially important for athletes with a negative energy balance, or for athletes with injuries, common endocrine imbalances, fatigue, and immunosuppression [66]. Furthermore, an adequate protein intake also decreases the presence of inflammation at a systemic level by decreasing the amount of circulating pro-inflammatory cytokines, regulating hormone levels such as GH and IGF-1, and strengthening the immune system [176,177]. Adequate protein intake should be considered, first and foremost, by considering individual needs as well as globally recommended guidelines [177]. Furthermore, maintaining an adequate fat intake is essential for correct cellular and tissue functioning; in particular, the intake of the omega-3 family should be increased in athletes [178]. Reducing the dose of dietary fat could compromise the immune system and antioxidant defenses by reducing the expression and production of cytokines [179]. By contrast, an increase in the dose of dietary fat could lead to an increase in the probability of the onset of cardiovascular pathologies [180]. Another aspect linked to the onset of OTS is an uncontrolled increase in oxidative stress with an increase in resting oxidative stress markers and an excess of free radicals. As a result, it is crucial for athletes to ensure adequate antioxidant needs [181]. For example, vitamins A, C, and E as well as minerals such as zinc, selenium, copper, and manganese are essential micronutrients that allow the body to fight against increased oxidative stress, naturally without excess as excessive intake of these micronutrients in the form of food supplements could lead to a negative effect on the body [182]. OTS is an overall body response to an excessive build-up of stressors, training, and lack of rest. While diet and particular dietary regimens are not the only factors determining the development of OTS, it is important to give due attention to nutrition’s role in the prevention and management of this condition. Therefore, a well-balanced diet in terms of energy, macronutrients, and micronutrients for an athlete could allow him or her to deal with the various stresses induced by training to favor adequate physiological adaptations and long-term performance. Finally, it is necessary to point out that, because this study is a narrative review, the articles included in it could be subject to selection errors, as a non-systematic approach was used to collect and analyze the articles. This is a possible limitation of this study, making further research and subsequent systematic reviews or meta-analyses necessary, given the importance of the topic covered and the fact that it still receives little attention, especially due to the negative effects of overtraining on human health. 

## 6. Conclusions

Training and performance improvement are key factors in a complex correlation between work overload and recovery time for an athlete’s physique. These factors, to improve performance, must be individualized for each athlete, considering their constant evaluation and a suitable treatment plan. If the physical stresses on an athlete are greater than the workload they can tolerate, imbalances could arise and lead to OTS. The importance of a timely diagnosis through the parameters and markers known to date with subsequent care of the athlete is clear in this study. In this context, nutrition can play a fundamental role, both in prevention and as a non-pharmacological treatment, with the aim of helping the athlete in reducing the likelihood of onset of OTS but also in its treatment, improving recovery and physical performance. Therefore, not only consultancy but also continuous updates and scientific research in the field of nutrition and food are essential for increasingly efficient support in the sports field.
